# Semi automated adjudication of vital sign alerts in step-down units

**DOI:** 10.1186/2197-425X-3-S1-A769

**Published:** 2015-10-01

**Authors:** M Fiterau, A Dubrawski, D Wang, L Chen, M Guillame-Bert, M Hravnak, G Clermont, E Bose, A Holder, A Murat Kaynar, D Wallace, MR Pinsky

**Affiliations:** Carnegie Mellon University, Auton Lab, Pittsburgh, United States; University of Pittsburgh, School of Nursing, Pittsburgh, United States; University of Pittsburgh, School of Medicine, Pittsburgh, United States; Department of Medicine, Emory University, Atlanta, United States

## Introduction

Machine Learning (ML) has shown predictive utility in analyzing vital sign (VS) data collected from physiologically unstable monitored patients. Training an ML model usually requires sizable amounts of labeled ground-truth data typically obtained via laborious manual chart reviews by expert clinicians.

## Objectives

To reduce effort of clinicians adjudicating vital sign alerts as true alerts or artifacts. The approach can also enable real time filtering of artifacts in vital sign monitoring systems.

## Methods

Noninvasive VS data including ECG-derived heart rate (HR), respiratory rate (RR), systolic and diastolic blood pressure (BP), and pulse oxygen saturation (SpO2) is monitored to issue alerts whenever VS exceed any of pre-set stability thresholds [1]. Two experts independently annotated 40 of such alerts only using informative low-dimensional projections of data onto statistical features extracted from raw VS data, automatically selected by our ML system. Then these experts adjudicated the same alerts using the available chart time series. We summarized the results to observe consistency of adjudication. The statistical features were extracted from each raw VS stream independently during the alert window. 260 such alerts were adjudicated using the framework described in [1] by a committee of 4 experts.

## Results

**Figure 1:**
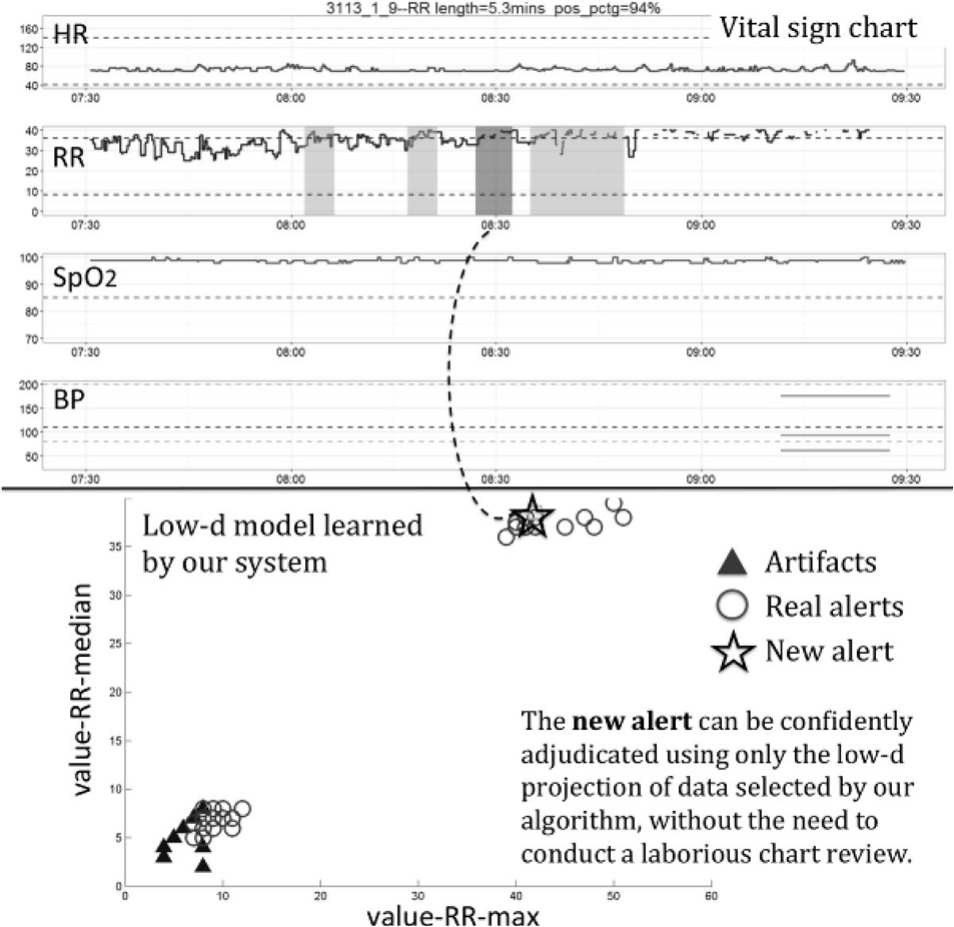
**SpO2 alert chart time and low-d model.**

Figure 1 shows an example of a clinical alert used in the study: VS chart (top) and low-dimensional projection recommended for adjudicating this alert by ML (below). In this projection, this alert can be confidently labeled as a true alert, which was later confirmed via chart review of the VS time series. Table 1 shows the outcome of the expert annotation, with 23 alerts adjudicated correctly using the low-dimensional projections without the need for chart review, only 5 requiring chart review, and 10 alerts on which the expert clinicians disagreed. In 2 of the cases, the intuition behind the ML-chosen projection did not agree with the chart review. The use of the ML model substantially reduced the need for manual chart reviews and overall data annotation effort (approximately twofold). The ML system was trained on 260 labeled samples and tested on a separate set of alerts. It adjudicated 75% of them with high confidence, and identified 32% of them as likely artifacts.

**Table 1:** Expert annotation study.

	Respiratory Rate	Oxygen Saturation
Correctly adjudicated using low-d projection	11	12
Required chart review to adjudicate	5	0
Chart review disagrees with low-d	1	1
Experts disagree	3	7

## Conclusions

Effective training of ML-based automatic alert adjudication systems can be achieved at substantial reduction of the effort required of expert clinicians. The resulting models can be used to confidently identify a significant percentage of the artifactual alerts.

## Grant Acknowledgment

NIH NINR R01NR013912; NSF1320347.
